
JOURNAL INFORMATION
==============================
NLM Title Abbreviation: Wirtschaftsdienst
Journal ID: Wirtschaftsdienst
NLM Journal ID: 101086612

Wirtschaftsdienst (Hamburg, Germany : 1949)

ISSN: 0043-6275

ARTICLE INFORMATION
==============================
PMCID: PMC8857624
PMID: 35221386
DOI: 10.1007/s10273-022-3105-8
Article ID: 3105
Article version: 1
Subjects: Zeitgespräch

Bürgergeld statt Hartz IV

Citizen’s Income (“Bürgergeld”) or Social Welfare (“Hartz IV”)?

Schäfer Holger schaefer.holger@iwkoeln.de

Institut der deutschen Wirtschaft Köln e.V., Georgenstraße 22, 10117 Berlin, Deutschland
Electronic publication date: 2022 Feb 19
Print publication date: 2022
Volume: 102
Issue: 2
First page: 82
Last page: 85
Copyright: © Der/die Autor 2022
License: Dieser Artikel wird unter der Creative Commons Namensnennung 4.0 International Lizenz veröffentlicht (creativecommons.org/licenses/by/4.0/deed.de).
Open Access wird durch die ZBW — Leibniz-Informationszentrum Wirtschaft gefördert.
License URL: https://creativecommons.org/licenses/by/4.0/

Keywords: J65, J68
issue-copyright-statement: © The Author(s) 2022

==============================

Mit dem „Bürgergeld“ unternimmt die Ampelkoalition einen erneuten Versuch, die Grundsicherung für Arbeitssuchende vom ungeliebten inoffiziellen Namen „Hartz IV“ zu lösen. Angesichts des negativen Images erscheint dies nachvollziehbar. Aber die Grundsicherung soll nicht nur einen neuen Namen bekommen. Im Koalitionsvertrag werden auch inhaltliche Änderungen in Aussicht gestellt – wenngleich vieles noch im Ungefähren bleibt.

## Schonvermögen

Eine dieser Änderungen betrifft die Anrechnung vorhandenen Vermögens und die Prüfung der Angemessenheit der Kosten der Unterkunft. Grundsätzlich ist im SGB II vorhandenes Vermögen für die Bemessung der Bedürftigkeit zu berücksichtigen. Dies soll verhindern, dass gegebenenfalls Menschen ohne laufendes Einkommen, aber mit großem Vermögen einen Anspruch auf Fürsorgeleistungen erhalten und reflektiert somit das Subsidiaritätsprinzip (Blömer et al., 2019a, 22): Hilfe sollen nur diejenigen erhalten, die sich nicht selbst helfen können.

Im Koalitionsvertrag wird in Aussicht gestellt, *erstens* in den ersten beiden Jahren des Bezugs von Bürgergeld keine Anrechnung des Vermögens und der Angemessenheit der Kosten der Unterkunft vorzunehmen und *zweitens* das Schonvermögen dauerhaft zu erhöhen. Die erste Maßnahme knüpft an eine Regelung an, die zu Beginn der Coronapandemie eingeführt wurde und die nach derzeitigem Stand noch bis zum 31. März 2022 gilt. Demnach wird für die Bewilligung der Grundsicherung Vermögen nicht berücksichtigt, sofern es nicht „erheblich“ ist, d. h. über einer Grenze von 60.000 Euro für das erste Mitglied einer Bedarfsgemeinschaft zuzüglich weiterer 30.000 Euro je zusätzlichem Mitglied liegt. Dabei erfolgt keine Prüfung, sondern es wird vermutet, dass kein erhebliches Vermögen vorliegt, wenn es Antragstellende so erklären (§ 67 SGB II). Diesem erleichterten Zugang zu Grundsicherungsleistungen lag die Idee zugrunde, dass im Zuge der Pandemie viele Menschen – insbesondere Kleinunternehmen und Soloselbstständige – erstmals und ausschließlich pandemiebedingt auf die Grundsicherung zurückgreifen müssen. Diesen wollte man „schnell und unbürokratisch“ helfen. Eine wesentliche Rolle spielte zudem der Erhalt der Arbeitsfähigkeit der Job-Center, da man mit einer erheblichen Steigerung der Zahl der Neuanträge rechnete (Deutscher Bundestag, 2020). Der gleiche Hintergrund besteht für die derzeit ebenfalls bis 31. März 2022 begrenzte Aussetzung der Prüfung der Angemessenheit der Kosten der Unterkunft.

Schon für die mehrfache Verlängerung der ursprünglich nur auf drei Monate begrenzten Erleichterungen erwies sich die Begründung nach kurzer Zeit als nicht mehr tragfähig. So stieg zwar im April 2020 die Zahl der Zugänge von erwerbsfähigen Leistungsbeziehenden in den Regelleistungsbezug sprunghaft von 105.000 auf 230.000. Doch war bereits im Juli 2020 wieder das Vorkrisenniveau erreicht. Im Winter 2020/2021 stiegen im Zuge der zweiten und dritten Pandemiewelle die Zugangszahlen zwar erneut stark an, verebbten aber ebenso schnell wieder und unterschritten im Mai 2021 das Niveau vom Mai 2019. Die Zahl der Neuanträge war mithin nur für einen sehr begrenzten Zeitraum erhöht. In den Bestandszahlen schlug sich die Pandemie kaum nieder. Im Dezember 2021 lag die Zahl der erwerbsfähigen Leistungsbeziehenden auf einem historischen Tiefstand (Statistik der Bundesagentur für Arbeit, 2022a).

Somit kann die Entlastung der Job-Center allenfalls in struktureller Hinsicht als Begründung für die avisierte zweijährige Aussetzung der Vermögensprüfung bzw. Prüfung der Kosten der Unterkunft herangezogen werden. Eine grundsätzliche Entlastung von derart aufwendigen und konfliktträchtigen Prüfungen kann vertretbar erscheinen, wenn die freiwerdenden Ressourcen für die individuelle Betreuung der Hilfebedürftigen eingesetzt werden. Es muss allerdings gegen die Frage abgewogen werden, ob die Grundsicherung ihren Charakter als nachrangige Leistung behält. Der Akzeptanz des Bürgergeldes würde es wenig helfen, wenn die Begünstigten im Zweifel über ein höheres Vermögen verfügen als diejenigen, die diese Leistung mit ihren Steuern finanzieren. Gleichsam wäre es der Akzeptanz abträglich, wenn Transferempfangenden Kosten der Unterkunft erstattet werden, die sich die Steuerzahlenden nicht leisten könnten. Im Falle der Unterkunftskosten erscheint – wenn es um eine Entlastung der Job-Center geht – die Pauschalierung von Leistungen als eine bessere Lösung gegenüber der temporären Abschaffung jeglicher Zugangsvoraussetzungen.

Die temporäre Aussetzung der Vermögensprüfung könnte für 9 % der Neuanträge eine andernfalls versagte Bewilligung eines Antrags auf Grundsicherung bedeuten. Hinzu kämen gegebenenfalls Personen, die wegen vorhandenen Vermögens erst gar keinen Antrag stellen. Profitieren würden vorrangig ältere Menschen mit beruflicher Qualifikation und langer Erwerbserfahrung (Beste et al., 2021). Somit könnte die Aussetzung der Vermögensanrechnung in Kombination mit der verlängerten Bezugsdauer von Arbeitslosengeld auf der einen Seite einen neuen Pfad zum vorzeitigen Ausscheiden aus dem Erwerbsleben eröffnen. Andererseits könnte durch die Maßnahme der Anreiz gestärkt werden, privat Vermögen zur Altersvorsorge zu bilden.

Die im Koalitionsvertrag darüber hinaus vorgesehene dauerhafte Erhöhung des Schonvermögens wird nicht dezidiert begründet. Offenbar liegt dem Vorschlag die Vorstellung zugrunde, es sei derzeit zu niedrig. Das nicht anzurechnende Vermögen setzt sich aus mehreren Komponenten zusammen: Grundfreibeträge und Freibeträge zur Altersvorsorge sind nach Geburtsjahr gestaffelt und sehen Mindest- und Höchstwerte vor. Hinzu kommt ein Freibetrag für Anschaffungen. Geförderte Altersvorsorge wie eine Riester-Rente bleibt komplett anrechnungsfrei. Ebenso werden eine selbst genutzte Immobilie und ein Kraftfahrzeug nicht angerechnet, wobei beide „angemessen“ sein müssen. Die Rechtsprechung veranschlagt im Regelfall dafür beim Kfz einen Verkehrswert von 7.500 Euro und bei der Immobilie eine Wohnfläche, die von der Haushaltsgröße abhängt und z. B. 80 qm für einen Einpersonenhaushalt oder 130 qm für einen Vierpersonenhaushalt beträgt. Daraus ergeben sich für verschiedene exemplarische Haushaltstypen die in Tabelle 1 dargestellten Schonvermögen. Inwieweit diese als „zu niedrig“ erscheinen, ist eine normative Frage. Das mittlere Nettovermögen einschließlich Immobilien beträgt für Haushalte, in denen der oder die Haupteinkommensbeziehende 50 bis 54 Jahre alt ist, 115.000 Euro (Niehues und Stockhausen, 2020). Mithin liegt das Schonvermögen zumindest für Mehrpersonenhaushalte in höherem Alter durchaus im Bereich mittlerer Vermögen.

Tabelle 1
**Nicht anzurechnendes Vermögen für verschiedene Haushaltstypen im SGB II**
in Euro

| | Allein-stehend, 40 Jahre | Paar ohne Kinder, jeweils 50 Jahre | Paar mit 2 Kindern, jeweils 35 Jahre |
|---|---|---|---|
| Grundfreibetrag | 6.000 | 15.000 | 16.700 |
| Altersvorsorgevermögen | 30.000 | 75.000 | 52.500 |
| Freibetrag für Anschaffungen | 750 | 1.500 | 1.500 |
| Summe | 36.750 | 91.500 | 70.700 |
| Zuzüglich geförderte Altersvorsorge in unbegrenzter Höhe | | | |
| Zuzüglich angemessenes Kfz und selbstgenutzte angemessene Immobilie | | | |

Quelle: eigene Darstellung.

## Sanktionen

Eine weitere Änderung betrifft die Sanktionen. Der Koalitionsvertrag betont, dass an Mitwirkungspflichten von Hilfebedürftigen festgehalten werden solle. Eine gesetzliche Neuregelung der Sanktionen, die durch das Urteil des Bundesverfassungsgerichts vom November 2019 erforderlich wurde, wird für dieses Jahr in Aussicht gestellt. Bis dahin solle ein einjähriges „Moratorium“ für Sanktionen gelten, wobei die genauen Umstände unklar bleiben. Derzeit werden Sanktionen im Vergleich zu den gesetzlichen Formulierungen in einer abgeschwächten Form verhängt. Im Hinblick auf das Urteil werden Meldeversäumnisse mit einer Kürzung des Regelsatzes um 10 % und darüber hinaus gehende Pflichtverletzungen mit einer Kürzung von 30 % des Regelsatzes sanktioniert. Selbst Fälle, in denen eine Mitwirkung vom Hilfebedürftigen in jeglicher Hinsicht verweigert wird, können somit derzeit mit einer Kürzung in Höhe von maximal 135 Euro sanktioniert werden – das entspricht einem Anteil von rund 15 % an der gesamten Transferleistung inklusive Kosten der Unterkunft eines Alleinstehenden.

Verschiedene Evaluationsstudien konnten Indizien für die Wirksamkeit von Sanktionen hinsichtlich des Übergangs in Beschäftigung finden. Bernhard et al. (2021, 11) fassen den Forschungsstand folgendermaßen zusammen: „Sanktionen entfalten bei den Betroffenen durch eine im Schnitt beschleunigte Aufnahme einer Erwerbstätigkeit intendierte Wirkungen.“ Allerdings ergeben sich auch Nebenwirkungen. So finden van den Berg et al. (2022) für junge Männer neben einer erhöhten Übergangswahrscheinlichkeit in Arbeit auch eine erhöhte Wahrscheinlichkeit für das Ausscheiden aus dem Arbeitsmarkt sowie einen Lohnabschlag. Wolf (2021) kann zudem zeigen, dass Sanktionen in der kurzen und mittleren Frist zwar einen positiven Effekt auf die Eingliederung in Beschäftigung haben, dieser sich aber in der längeren Frist ab etwa 30 Monaten umkehrt. Insofern ergebe sich ein Zielkonflikt zwischen schneller und nachhaltiger Integration in den Arbeitsmarkt.

Aus den Befunden der Wirkungsforschung kann eine Forderung nach Abschaffung der Sanktionen nicht abgeleitet werden. Sie sind ein Instrument, mit dem die unmittelbare Integration in Beschäftigung erleichtert wird. Darüber hinaus sind sie ein wichtiger Ausdruck des Prinzips der Reziprozität in der Grundsicherung: Die Gesellschaft hilft denen, die sich nicht selbst helfen können. Im Gegenzug schulden Hilfeempfangende im Rahmen ihrer Möglichkeiten das Bemühen, künftig ohne diese Hilfe auszukommen. Sollte das im Koalitionsvertrag avisierte Moratorium also eine vollständige vorübergehende Aussetzung der Sanktionen bedeuten, müsste mit einer Verringerung der Übergänge aus dem Transferbezug in Arbeit gerechnet werden. Gegebenenfalls leidet auch die gesellschaftliche Akzeptanz. Es spricht wenig dagegen, die gegenwärtige, ohnehin sehr begrenzte Sanktionspraxis mindestens beizubehalten. Überlegenswert wäre, den Spielraum des Verfassungsgerichtsurteils in der Hinsicht zu nutzen, als dass es eine Totalsanktion für die Verweigerung einer Erwerbstätigkeit ohne wichtigen Grund durchaus ermöglichen würde, da es in diesem Fall an den Voraussetzungen für Hilfebedürftigkeit fehle (Bundesverfassungsgericht, 2019, Rz 209). Für die gesetzliche Neuregelung der Sanktionen wäre zu berücksichtigen, dass die integrationsfördernde Wirkung der Sanktionen erhalten bleibt. Der negativen Nebenwirkung einer geringeren Beschäftigungsstabilität könnte mit einem erweiterten Coaching entgegengewirkt werden.

## Erwerbsfreibetrag

Eine dritte Änderung betrifft den Erwerbsfreibetrag, der bestimmt, inwieweit Erwerbseinkommen auf Transferleistungen angerechnet werden. Die Ampelkoalition beabsichtigt, die Zuverdienstmöglichkeiten zu „verbessern" und dabei Anreize für eine sozialversicherungspflichtige Beschäftigung zu steigern, indem Grenzbelastungen von 100 % ausgeschlossen werden. Die genauen Parameter einer neuen Regelung bleiben vorerst offen.

Die geltende Regelung sieht einen Grundfreibetrag von 100 Euro vor. Für Bruttoeinkommen über 100 bis 1.000 Euro werden 20 % nicht angerechnet, für darüber hinausgehende Einkommen gilt ein Freibetrag von 10 %. Der Freibetrag wird bei 1.200 Euro bei Hilfebedürftigen ohne Kinder bzw. 1.500 Euro bei solchen mit Kindern gedeckelt. Die Regelung begünstigt in starkem Maße die Kombination von Transferbezug und einer Beschäftigung mit geringer Stundenzahl und entsprechend geringem Erwerbseinkommen auf Kosten von Beschäftigungsverhältnissen mit höherer Stundenzahl. In der Folge liegt das zusätzliche verfügbare Einkommen je zusätzlicher Stunde Arbeit bei einer Ausweitung der Arbeitszeit von Teilzeit zu Vollzeit bei rund einem Euro, teilweise noch darunter (Schäfer, 2019). Dies ist von erheblicher Bedeutung, da für das verfügbare Einkommen der Aufstockenden nicht erheblich ist, wie viel Nettoeinkommen aus einem gegebenen Bruttoeinkommen resultiert. Ausschlaggebend ist allein der Erwerbsfreibetrag.

Es bestehen mithin nur schwache finanzielle Anreize für Hilfebedürftige, mehr als eine Teilzeitbeschäftigung auszuüben, obgleich das Ziel der Sozialpolitik darin bestehen sollte, dass der eigene Bedarf zu einem möglichst großen Teil aus eigener Kraft erwirtschaftet wird. Die Anreizstruktur bleibt nicht ohne Wirkung. Im Juni 2021 waren nur 12 % der Aufstockenden sozialversicherungspflichtig vollzeitbeschäftigt, dagegen übten 30 % lediglich eine geringfügige Beschäftigung aus (Statistik der Bundesagentur für Arbeit, 2022b). Der Reformbedarf ist vor diesem Hintergrund offenkundig.

Eine simple Erhöhung der Freibeträge löst zwar das Problem der geringen Anreize, ruft dafür aber neue Probleme hervor. *Erstens* gehen höhere Freibeträge mit fiskalischen Kosten einher. *Zweitens* vergrößert sich der Kreis der Anspruchsberechtigten, weil sich die Einkommensgrenze, bis zu der ein Anspruch auf ergänzende Leistungen besteht, nach oben verschiebt. Im Ergebnis könnte eine solche Reform zu mehr Leistungsempfangenden führen. *Drittens* erhalten Erwerbstätige mit einem Einkommen knapp oberhalb der zur Aufstockung berechtigenden Grenze einen Anreiz zur Reduzierung ihres Arbeitsangebots. Denn ein Teil des mit der Arbeitszeitreduzierung einhergehenden Einkommensverlustes wird durch die Aufstockung kompensiert. Je mehr die Grenze an mittlere Einkommen heranreicht, desto größer ist die Zahl der betroffenen Beschäftigten. Statt einer Erhöhung erscheint daher eine Umgestaltung des Freibetrags sinnvoller.

Die Problematik ist keineswegs neu. Bereits 2010 untersuchte der Sachverständigenrat die fiskalischen und die Beschäftigungswirkungen von insgesamt 972 Reformszenarien mit Hilfe eines Mikrosimulationsmodells. Dabei können die Modelle kaum alle drei Zielvariablen Partizipation, Arbeitsvolumen und fiskalische Belastung gleichzeitig optimieren. Es zeigte sich indes, dass eine Beschneidung des Freibetrags für geringe Einkommen unter Ausweitung des Freibetrags für höhere Einkommen die Ziele am besten miteinander vereinbaren kann (SVR 2010, 306 ff.). Operationalisiert ist diese Erkenntnis unter anderem in mehreren aktuellen Reformvorschlägen (Bruckmeier et al., 2018; Blömer et al., 2019b; Schäfer, 2019). Ihnen gemeinsam ist, dass der Grundfreibetrag stark vermindert wird oder ganz wegfällt, sich danach ein Einkommensbereich mit einer Vollanrechnung anschließt, um schließlich für höhere Einkommen den Freibetrag ausweiten zu können. Im Ergebnis wird der bisher konkave Einkommensverlauf zu einem konvexen. Kleine Einkommen, wie sie aus Minijobs resultieren, werden stärker angerechnet. Höhere Einkommen, wie sie aus Vollzeittätigkeiten resultieren, werden weniger stark angerechnet als im Status quo.

Angesichts der offenkundigen Fehlanreize des bestehenden Erwerbsfreibetrages könnte eine solche Reform das Volumen des Arbeitskräfteangebots stärken. Die Befunde diverser Mikrosimulationen legen allerdings nahe, dass der Effekt eher überschaubar bleibt. Daher wird eine Verbesserung der Anreizstruktur das Problem allein nicht lösen können. Erforderlich bleiben auch weiterhin aktivierende Ansätze der Arbeitsmarktpolitik durch die Job-Center.

Germany’s new government attempts once again to get rid of the infamous “Hartz IV” name for the country’s basic income support. The new “Bürgergeld” not only sports a new name, it also formulates new rules for sanctions and for considering income and property.

Holger Schäfer ist Senior Economist am Institut der deutschen Wirtschaft Köln e. V. in Berlin.


Literatur

Bernhard, S., M. Bossler, T. Kruppe, T. Lietzmann, M. Senghaas, G. Stephan, S. Trenkle, J. Wiemers und J. Wolff (2021), Vorschläge zur Reform der Grundsicherung für Arbeitsuchende und weiterer Gesetze zur sozialen Absicherung, IAB-Stellungnahme, 5/2021.
Beste, J., M. Trappmann und J. Wiederspohn (2021), Vereinfachter Zugang zur Grundsicherung: Wer von einer Schonfrist bei Vermögensanrechnung und Aufwendungen für die Unterkunft profitieren würde, IAB-Forum, 13. Dezember, https://www.iab-forum.de/verein-fachter-zugang-zur-grundsicherung-wer-von-einer-schonfrist-beivermoegensanrechnung-und-aufwendungen-fuer-die-unterkunft-profitieren-wuerde/ (18. Januar 2022).
Blömer M Fuest C Peichl A Die Hartz 4-Reformdebatte ifo-Schnelldienst 2019 72 6 21 25
Blömer M Fuest C Peichl A Raus aus der Niedrigeinkommensfalle(!) Der ifo-Vorschlag zur Reform des Grundsicherungssystems ifo-Schnelldienst 2019 72 4 34 43
Bruckmeier, K., J. Mühlhan und J. Wiemers (2018), Erwerbstätige im unteren Einkommensbereich stärken. Ansätze zur Reform von Arbeitslosengeld II, Wohngeld und Kinderzuschlag, IAB-Forschungsbericht, 9/2018.
Bundesverfassungsgericht (2019), Leitsätze zum Urteil des Ersten Senats vom 5. November 2019 — 1 BvL 7/16 — (Sanktionen im Sozialrecht), https://www.bundesverfassungsgericht.de/SharedDocs/Entscheidungen/DE/2019/11/ls20191105_1bvl000716.html (20. Januar 2022).
Deutscher Bundestag (2020), Entwurf eines Gesetzes für den erleichterten Zugang zu sozialer Sicherung und zum Einsatz und zur Absicherung sozialer Dienstleister aufgrund des Coronavirus SARS-CoV-2 (Sozialschutz-Paket), Drucksache 19/18107.
Niehues, J. und M. Stockhausen (2020), Vermögensgrenzen: große gruppenspezifische Unterschiede, IW-Kurzbericht, 105/2020.
Schäfer, H. (2019), Einkommen aus Erwerbstätigkeit und SGB II-Leistungen, Kurzgutachten für die Initiative Neue Soziale Marktwirtschaft.
Statistik der Bundesagentur für Arbeit (2022a), Berichte: Analyse Arbeitsmarkt, Grundsicherung für Arbeitsuchende, Dezember.
Statistik der Bundesagentur für Arbeit (2022b), Erwerbstätige erwerbsfähige Leistungsberechtigte, Januar.
SVR — Sachverständigenrat zur Begutachtung der wirtschaftlichen Entwicklung (2010), Chancen für einen stabilen Aufschwung, Jahresgutachten 2010/11.
Van den Berg, G. J., A. Uhlendorff und J. Wolff (2022), The Impact of Sanctions for Young Welfare Recipients on Transitions to Work and Wages, and on Dropping Out, Economica, 89, Januar, 1–28.
Wolf, M. (2021), Schneller ist nicht immer besser: Sanktionen können sich längerfristig auf die Beschäftigungsqualität auswirken, IAB-Forum, 24. Juni, https://www.iab-forum.de/schneller-ist-nicht-immer-besser-sanktionen-koennen-sich-laengerfristig-auf-die-beschaeftigungs-qualitaet-auswirken/ (20. Januar 2022).
